# The Role of C/EBP‐Homologous Protein in Idiopathic Inflammatory Myopathies

**DOI:** 10.1111/jcmm.70919

**Published:** 2025-10-29

**Authors:** Monica Sciacco, Patrizia Ciscato, Letizia Bertolasi, Maria Guttuso, Stefania Corti, Deborah Mattinzoli, Masami Ikeata, Giuseppe Castellano, Giacomo Pietro Comi, Simona Zanotti

**Affiliations:** ^1^ Neuromuscular and Rare Disease Unit Fondazione IRCCS Ca' Granda Ospedale Maggiore Policlinico Milan Italy; ^2^ Neurology Unit Fondazione IRCCS Ca' Granda Ospedale Maggiore Policlinico Milan Italy; ^3^ Renal Research Laboratory Fondazione IRCCS Ca' Granda Ospedale Maggiore Policlinico Milan Italy; ^4^ Department of Clinical Sciences and Community Health University of Milan Milan Italy; ^5^ Unit of Nephrology, Dialysis and Renal Transplantation Fondazione IRCCS Ca' Granda Ospedale Policlinico Milan Italy; ^6^ Dino Ferrari Centre, Department of Pathophysiology and Transplantation (DEPT) University of Milan Milan Italy

**Keywords:** CD206, CHOP, EndoMT, ER stress, inflammatory myopathy, necrosis

## Abstract

The Idiopathic Inflammatory Myopathies (IIMs) are a group of autoimmune disorders characterised by persistent muscle inflammation and diverse clinical manifestations. Common symptoms include muscle weakness, myalgia, and elevated serum creatine kinase levels. Recent findings highlight the relevance of muscle fibre necrosis in IIMs. We therefore grouped our IIM patients according to the percentage of necrotic fibres in muscle biopsy. Our clustered patients were analysed for the inflammatory milieu, the capillary network, and the endoplasmic reticulum stress. In patients with a higher percentage of necrotic fibres we detected a more marked presence of CD206 positive cells, an activation of the endothelial–mesenchymal transition process, an altered capillary network, more marked ER stress and connective tissue deposition. Furthermore, our study revealed a key role of C/EBP‐homologous protein (CHOP), a multifunctional transcription factor that contributes to cellular functions including apoptosis, autophagy, inflammation, mediation of ER stress and induction of fibrosis. Our study suggests that CD206 positive cells and CHOP have an important role in pathogenetic mechanisms and could therefore be considered possible therapeutic targets to modulate the inflammatory response of these patients, namely to contain or slow down the progression of fibrosis.

**Abbreviations**
ATF6: Activating transcription factor 6
Bcl‐2: B‐cell lymphoma 2
Bip: immunoglobulin heavy chain binding protein
CHOP: C/EBP homologous protein
ER: endoplasmic reticulum
IIM: idiopathic inflammatory myopathy
IRE1: Inositol‐requiring protein 1
LC3: microtubule‐associated protein 1 light chain 3 (MAP1LC3, referred to as LC3)
MHC‐I: Major Histocompatibility Complex class I
PERK: Protein Kinase RNA‐like ER Kinase
UPR: Unfolding protein response
XBP1: X‐box‐binding protein

## 1 Introduction

Idiopathic inflammatory myopathies (IIMs), generally indicated as myositis, refer to a diverse set of autoimmune disorders characterised primarily by persistent muscle inflammation, accompanied by various clinical manifestations affecting skin, lungs and joints, along with the production of autoantibodies. Currently, the classification of IIMs recognises five main types: polymyositis (PM), dermatomyositis (DM), anti‐synthetase syndrome (ASS), immune‐mediated necrotizing myopathies (IMNM) and inclusion‐body myositis (IBM) [1]. These categories are quite distinct in terms of prognosis, treatment response, clinical manifestations and organ involvement, indicating different underlying pathophysiological mechanisms. Diagnosis relies on a combination of clinical symptoms, muscle biopsy features, MRI patterns, serological assessments and serum levels of muscle enzymes.

Common symptoms include proximal muscle weakness in both upper and lower limbs, myalgia, and elevated serum creatine kinase (CK) levels, except for IBM in which CK values are relatively lower. IMNM exhibits a more rapid and severe disease progression compared to DM and PM, characterised by very high CK levels and a distinct histological pattern in muscle biopsies [2]. When biopsies are not feasible or inconclusive, advanced MRI techniques can offer supportive evidence by identifying characteristic patterns of inflammation, edema, and fatty infiltration [3].

Histologically, PM shares similarities with IBM, including endomysial infiltration of CD8 and diffuse MHC class I upregulation in muscle fibres. DM patients typically show perifascicular atrophy, MHC class I upregulation, and complement deposition in the endomysial microvasculature, accompanied by skin lesions such as heliotrope rash and Gottron signs.

The growing need for improved histological characterisation of these patients has led to the identification of potential diagnostic markers [4, 5].

Treatment primarily includes high doses of glucocorticoids in combination with other immunosuppressive drugs, but responses to therapy are highly variable; hence the need for new therapeutic approaches. Besides inflammatory pathways, non‐immune mechanisms contribute to IIMs, i.e., endoplasmic reticulum stress (ER), protein aggregation, NF‐κB pathway activation, free radical production and necroptosis [6, 7, 8, 9]. Mitochondrial abnormalities, associated with chronic inflammation and oxidative stress, are also observed in IIM myopathies.

The crosstalk among inflammatory cells, muscle and endothelial cells is crucial in IIM pathogenesis, leading to alterations in microvascular networks. Capillary abnormalities, including micro‐vasculopathy, are reported in DM and IMNM patients. Endothelial–mesenchymal transition (EndoMT) is implicated in these capillary alterations, influenced by pro‐inflammatory cytokines, growth factors, and oxidative/shear stress.

Myopathic changes as fibre calibre variability, centronucleated and splitting fibres and variable degrees of fibrosis are typically detected in IIM muscle biopsy [10].

The ER stress response plays a crucial role in regulating the unfolded protein response (UPR) signalling in various tissues, including skeletal muscle. The ER is an endomembrane system involved in the synthesis, folding, and modification of proteins. In case of imbalance between the protein‐folding load and the capacity of the ER to handle it, ER stress can occur. The UPR is a cellular signalling pathway that is activated in response to ER stress. The UPR aims at restoring ER homeostasis by adjusting the protein‐folding capacity of the ER and decreasing the load of unfolded proteins. The UPR involves the activation of three main transmembrane proteins: IRE1 (Inositol‐Requiring Enzyme 1), PERK (Protein Kinase RNA‐like ER Kinase), and ATF6 (Activating Transcription Factor 6). These proteins initiate a series of signalling events that lead to changes in gene expression and cellular processes. In muscle tissues, the UPR is particularly important because of the high demand for proper protein folding and synthesis, especially during periods of increased muscle activity or stress. Conditions such as muscle injury, exercise, and certain diseases can induce ER stress in muscle cells, triggering the UPR to restore normal ER function and maintain cellular homeostasis. ER stress‐induced autophagy pathways are implicated in IIMs, including IMNM [11], potentially playing a role in both muscle damage and restoration. Recent studies suggest a close association between ER stress‐mediated autophagy activation and various physiological and pathological processes in IIM muscle biopsies, with correlations to muscle weakness in IMNM.

Previous studies have provided evidence of ER stress response activation in polymyositis, DM [12, 13] and sIBM [14], which also resulted in impaired autophagy. However, the functional role of ER stress in these conditions remains poorly understood.

Among the downstream effectors of UPR, the C/EBP homologous protein (CHOP) plays a central role in promoting apoptosis during prolonged or unresolved ER stress [15]. CHOP, a stress‐inducible transcription factor, is mainly activated via the PERK‐eIF2α‐ATF4 pathway and contributes to myofiber damage by inducing cell death and inhibiting protective mechanisms like autophagy [16]. Its roles include the regulation of apoptosis, modulation of protein synthesis via the PERK‐eIF2α‐ATF4 axis, and influence on cellular redox balance through downstream effectors such as GADD34 and ERO1α [17]. Increased CHOP expression has been observed in muscle biopsies from IIM patients [18]. While immune mechanisms are central, accumulating evidence suggests that non‐immune cellular stress pathways, including ER stress and UPR activation, contribute to muscle damage, dysfunction and possibly repair processes in IIM. Given CHOP's central position as a mediator of stress‐induced apoptosis and its capacity to amplify ER stress responses, it is plausible that CHOP might play a key role in the pathogenesis of IIM, both in driving myofiber injury and influencing the balance between damage and regeneration.

This study focuses on muscle biopsies from patients diagnosed with inflammatory myopathy showing a variable degree of necrotic fibres and characterised by different inflammatory profiles. After clustering our patients into three groups based on the percentage of necrotic fibres, we analysed the capillary network and the ER stress response. Our aim is to better define the molecular mechanisms underlying inflammatory myopathies for the identification of possible new therapeutical targets and improvement of patient outcome.

## 2 Materials and Methods

### 2.1 Patients

This study was conducted on 30 patients and 18 age‐matched controls, the latter being individuals who had undergone skeletal muscle biopsy for suspected neuromuscular disease, but whose biopsy had turned out normal. All patients were recruited from the Neurology–Neuromuscular and Rare Disease Unit of Fondazione IRCCS Ca' Granda Ospedale Maggiore Policlinico of Milano.

Skeletal muscle biopsy was taken at the biceps brachii in all but three patients in whom quadriceps muscle was selected. All patients managed to undergo muscle biopsy soon after the clinical diagnosis, therefore prior to initiating pharmacological treatment with steroids. The study was conducted in compliance with the ethical standards outlined in the Declaration of Helsinki, as well as national legislation and institutional guidelines. All participating subjects provided written informed consent, which had been approved by the local Ethical Committee, to undergo skeletal muscle biopsy, blood sampling as well as storage, analysis, and collection of their data, including clinical information. The study protocol has also been approved by the local Ethical Committee.

### 2.2 Clinical Aspects

No statistically significant difference was detected in average age at muscle biopsy between patients (average age 54.7 ± 3.54 years) and controls (average age 50.6 ± 4.88 years). Both sexes were represented in the patient group, with a predominance of females (18F/12M). The control subject group included eight females and ten males.

We stratified the patients into three distinct groups based on the percentage of necrotic fibres detected at muscle biopsy. This stratification aimed to evaluate whether varying degrees of fibre necrosis could differentially influence the inflammatory components, such as the capillary network and the ER stress response. Positivity for MHC‐I was detected in all samples. Muscle biopsies from controls did not show any necrotic fibres or immunoreactivity for MHC‐I.

Clinically, all patients reported skeletal muscle weakness. Cramps were more often referred to by patients in Group 1, whereas myalgias were similarly reported by Group 1 and Group 3 patients. Myoglobinuria and dysphagia were equally represented in all three groups. Limb weakness was mostly proximal and more marked in patients from Group 2 (83.3%) followed by those from Group 3 (62.5%). Extra muscular clinical manifestations involved skin in 6/30 patients (20%), heart in 3/30 patients (10%) and lung in 3/30 (10%); malignancy was reported in two patients (6.67%). In patients who had undergone electromyography (EMG), the pattern was predominantly myopathic.

Serum creatine phosphokinase (CK) levels were increased in all patients, values ranging from 380 IU/L to 46,890 IU/L (average values 7429 ± 1824 IU/L). More in detail, CK reference values were 7466 ± 1640 IU/L in Group 1, 9872 ± 4266 IU/L in Group 2 and 3717 ± 1413 IU/L in Group 3.

Demographic, clinical, and laboratory profiles of patients and controls are summarised in Table 1.

**TABLE 1:** Demographic, clinical and laboratory data of patients and controls.

Demography	Group 1	Group 2	Group 3	Total	Ctrl	1 vs. 2	1 vs. 3	2 vs. 3
						*p*	*p*	*p*
*N*° subjects	*N* = 10	*N* = 12	*N* = 8	*N* = 30	*N* = 18			
Female	6 (60%)	9 (75%)	3 (37.5%)	18 (60%)	8 (44.4%)			
Caucasian	9 (90%)	12 (100%)	7 (87.5%)	28 (93.3%)	18 (100%)			
Age at biopsy (years)	59.3 ± 5.90	59.6 ± 5.06	41.7 ± 6.74	50.65 ± 4.88	50.6 ± 4.88			
Biopsy‐onset of symptoms (months)	5.0 ± 3.84	3.9 ± 2.62	3.5 ± 2.7	4.2 ± 3.29				
Clinical traits								
Hyposthenia	10 (100%)	12 (100%)	8 (100%)	30 (100%)				
Cramps	9 (90%)	2 (16.6%)	2 (25.0%)	13 (26%)				
Myalgias	8 (80%)	5 (41.6%)	7 (87.5%)	20 (66.6%)				
Myoglobinuria	2 (20%)	4 (33.3%)	1 (12.5%)	7 (23.3%)				
Dysphagia	2 (20%)	3 (25.0%)	2 (25.0%)	7 (23.3%)				
Fatigability/weakness of proximal limbs	2 (20%)	10 (83.3%)	5 (62.5%)	17 (56.6%)				
Skin lesions	2 (20%)	1 (8.33%)	3 (37.5%)	6 (20%)				
Heart	2 (20%)	0	1 (12.5%)	3 (10%)				
Lung	2 (20%)	0	1 (12.5%)	3 (10%)				
Laboratory data								
Creatine phosphokinase (U/L)	7466 ± 1640	9872 ± 4226	3717 ± 1413	7429 ± 1824	6–190	0.174	0.083	0.373
Autoantibodies								
Jo‐1	2	0	1					
HMGCR	1	2	1					
Seronegative	1	1	1					
ANA	0	2	2					
AMA‐M2	1	1	0					
PM/Slc	0	1	0					
N.D.	5	5	3					

Abbreviations: AMA‐M2, Anti‐mitochondrial M2 antibody; ANA, antinuclear antibody; HMGCR, 3‐hydroxy‐3‐methyl‐glutaryl‐coenzyme A reductase; Jo‐1, histidyl‐tRNA synthetase; ND, not determined. (% frequency); PM/Slc, anti‐polymyositis/scleroderma.

### 2.3 Muscle Biopsy

Routine Haematoxylin and Eosin (H&E) and Modified Gomori Trichrome (MGT) histological stains were performed on 8 μm‐thick cryostat muscle sections. The number of necrotic fibres was counted on MGT‐stained muscle sections and the percentage of necroses was calculated considering the number of fibres in each field. On the H&E‐stained muscle sections, the fibrotic area (connective and adipose tissue) was quantified using Leica Application Suite 4.9.0 and ImageJ 1.53c (https://imagej.nih.gov/ij/download.html) software [19]. The percentage of centronucleated and splitting fibres was also evaluated (H&E). For all analyses, on each section, four randomly, selected, non‐overlapping fields were photographed at 20× magnification, using optical microscope Leica DC200 equipped with camera and IM50 image analysis software (Leica Microsystems, Wetzlar, Germany).

Morphological analysis was completed with the staining for myosin ATPase (pH 9.4–4.6), cytochrome c oxidase (COX), succinate dehydrogenase (SDH), acid phosphatase (AP), NADH dehydrogenase, Oil Red O and Periodic Acid‐Schiff (PAS).

The percentage of fibres with total or partial COX deficiency was evaluated on four randomly, selected, non‐overlapping fields of the COX‐stained sections photographed at 20× magnification. The percentage of acid phosphatase positive fibres was similarly evaluated. Cross‐sectional area (CSA) of type I and type II fibres was measured on pH 4.6 myosin ATPase‐stained sections. CSA was assessed by manually drawing the perimeter of each fibre and by calculating the corresponding area (μm^2^) using ImageJ 1.53c software. Only cross‐section fibres were included in the analysis.

### 2.4 Morphometric Indexes of Muscle Capillarization

8 μm‐thick muscle cryosections were fixed for 5 min in cold acetone/methanol (1:1), washed three times with Phosphate Buffer Saline (PBS) and stained with Ulex Europaeus Agglutinin I (UEA‐I), fluorescein isothiocyanate–conjugated (Vector Laboratories, Burlington, ON, Canada) diluted 1:100 in PBS for 40 min at room temperature (RT). Capillary and muscle fibre counts were performed using ImageJ 1.53c (https://imagej.nih.gov/ij/download.html) on four randomly non‐overlapping fields acquired at 20× magnification with a Leica fluorescence microscope equipped with camera DFC420C (Leica, Wetzlar, Germany). Leica Application Suite V4.6.2 software was used for image acquisition.

Analysis of capillary profiles included the evaluation of capillary contacts (CC), the sharing factor (SF), the individual capillary to fibre ratio (C/Fi), the capillary to fibre perimeter exchange index (CFPE), the capillary density, and the number of capillaries/muscle fibre (FA/C), on at least 40 single fibres randomly selected in each image [20]. All transversely cut capillaries were counted. If a capillary was sectioned longitudinally it was excluded from the count [21]. To ensure that analysis of capillary density was not overestimated because of muscle atrophy, capillary density was also evaluated as a function of the number of muscle fibres in the section (capillaries/muscle fibres ratio). Sequential estimation analyses indicate that 50 fibres from one biopsy are sufficient to characterise capillary parameters [22].

### 2.5 Immunofluorescence

For immunofluorescence staining muscle 8 μm‐thick cryosections were fixed with acetone for 3 min, washed three times with PBS and blocked with 1% normal goat serum (Vector Laboratories) diluted in PBS for 30 min at RT. Primary antibody was incubated overnight at 4°C. Following three washes with PBS, muscle sections were incubated with the appropriate secondary antibody Alexa‐488 or Alexa‐594 (1:1000; Thermo Fisher, Waltham, MA, USA) for 2 h at RT. Finally, after three washes with PBS, slides were mounted with anti‐fading reagent Fluormount (Thermo Fisher); DAPI was used for counterstaining of nuclei. Caveolin‐3 (1:1000, mouse monoclonal; BD Transduction Laboratories, Franklin Lakes, NJ, USA) or Laminin‐α2 (1:1000 rabbit polyclonal; Sigma, St. Louise, MO, USA) were used for sarcolemma staining.

For inflammatory markers, muscle sections were each incubated with one of the following antibodies: MHC class I (MHC‐1, mouse monoclonal 1:100; Dako, Glostrup, Denmark), CD68 (mouse monoclonal, 1:100; Dako), CD206‐FITC conjugated (mouse monoclonal, 1:50; Miltenyi Biotec, Bergisch Gladbach, Germany), CD8, CD4 (both mouse monoclonal, 1:100; Dako, Glostrup, Denmark), and NF‐kB p65 (rabbit polyclonal, 1:400; Cell Signalling, Danvers, MA, USA).

The positivity staining for MHC‐1 was evaluated by visual inspection of immunofluorescence muscle sections and graded as myofiber sarcolemma and cytoplasm positivity.

Signal positivity and muscle fibre counts were performed using ImageJ 1.53c on four randomly non‐overlapping fields acquired at 20× magnification with a Leica fluorescence microscope equipped with camera DFC420C (Leica). Leica Application Suite V4.6.2 software was used for image acquisition. Quantification of CD68, CD206, CD8 and CD4 was performed by counting individually in each of the four acquired fields and normalised by the number of fibres in each field and expressed as a percentage of CDs positive cells/number of myofibres.

For EndoMT markers, muscle sections were each incubated with one of the following primary antibodies: VE‐Cadherin (rabbit monoclonal, 1:100, Sigma), CD31/PECAM (mouse monoclonal, 1:100; Millipore), α‐smooth muscle actin (α‐SMA, mouse monoclonal, 1:200, Sigma), fibronectin (rabbit polyclonal, 1:200; ThermoFisher) and fibroblast‐specific protein‐1 (FSP1, mouse monoclonal, 1:150; Sigma).

For ER stress markers the following primary antibodies were tested: CHOP (1:100, rabbit monoclonal, Sigma), calreticulin (rabbit polyclonal, 1:100, ThermoFisher), XBP‐1 (1:100) rabbit polyclonal (Novus Biologicals), Grp78/BiP (1:100), ATF6 (1:100) and P‐PERK (1:100) all rabbit polyclonal from Abcam (Cambridge, UK).

### 2.6 Tissue Processing for MACSima Platform

The MACSima Imaging System (Miltenyi Biotec, Bergisch Gladbach, Germany) is an entirely automated platform that integrates liquid handling and widefield microscopy for repetitive immunofluorescence imaging.

Human muscle sections, frozen to a thickness of 8 μm, were placed onto a Super Frost plus slide and fixed using cold acetone for 3 min at −20°C. Following a brief air drying, the slide was kept at −80°C for a minimum of 1 day. Right before the experiment, the slide was re‐fixed using cold acetone at −20°C for 10 min, then air‐dried at RT for 30 min, and subsequently placed onto a MACSwell TM Four Imaging Frame (130‐124‐676, Miltenyi Biotec) where it was washed three times with MACSima Running Buffer (Miltenyi Biotec) prior to initial DAPI pre‐staining at a 1:5 ratio (130‐127‐574, Miltenyi Biotec) for 20 min. The samples were subsequently washed three times using Running Buffer, concluding with a final sample volume of 475 μL in the Running Buffer. The antibodies labelled with fluorochrome, including CD206‐APC (REAL518), CD68‐PE (REA1306), CD31‐FITC (REA1028), and Collagen type I‐APC (REAL958) at a dilution of 1:50 were set up in a MACSwell Deepwell Plate (130‐126‐865, Miltenyi Biotec) using MACSima Running Buffer, and subsequently sealed with MACSwell Sealing Foil (130‐126‐866, Miltenyi Biotec) to avoid evaporation. The obtained image datasets were employed for the following image processing conducted in MACS iQ View Software Version 1.3.2 (Miltenyi Biotec).

### 2.7 Western Blot

Western blotting analysis was performed on muscle homogenates from selected patients and controls. 30 μg of sample was electrophoresed on Bolt Bis‐Tris‐Plus Mini Protein Gels (gradient 4%–12%; ThermoFisher) and transferred onto LF‐PVDF membranes (Bio‐Rad, Hercules, CA, USA). Membranes were probed with ER stress/UPR antibody pack (Novus Biologicals) containing the following antibodies: GRP78 (1:2000), XBP1 (1:1000), CHOP (1:5000), IRE1‐α (1:1000), IRE1‐α (Ser‐724) (1:700) all rabbit polyclonal; ATF6 (1:700) mouse monoclonal, calreticulin (1:1000, rabbit polyclonal, ThermoFisher) and NF‐kB p65 (rabbit polyclonal, 1:1000, Cell Signalling).

Membranes were probed with the apoptotic markers Bcl‐2 (1:1000, rabbit polyclonal, Abcam, Cambridge, UK) and with autophagic markers p62 (1:700, rabbit polyclonal, Millipore, Burlington, MA, USA) and LC3B (1:1000, Cell Signalling).

Actin (monoclonal antibody, 1:2000 Sigma Aldrich) was used as an indicator of protein loaded. The membranes were incubated with rabbit or mouse‐HRP secondary antibodies diluted 1:2000 for 75 min at RT (ThermoFisher). Immunoreactive bands were developed with Super Signal West Femto Substrate (ThermoFisher) for 5 min at RT. Acquisition of immunobands was performed by ODYSSEY LI‐COR Model 2800 and quantitated densitometrically using n‐Image Studio Software (Li‐COR Biosciences, Bad Homburg, Germany). For each sample, the values (Arbitrary Units) of the protein markers were normalised to the corresponding values of actin signal to account for loading differences. The normalised values from individual patients and controls were then statistically analysed using GraphPad Prism.

### 2.8 Statistical Analysis

Statistical analysis was performed using GraphPad Prism 10.1.0 (GraphPad Software Inc., LaJolla, CA, USA). Clinical and morphological data are expressed as mean ± SEM. Data were tested for normality using the Kolmogorov‐Smirnov test before performing the ANOVA test. A two‐tailed unpaired *t*‐test with a confidence interval of 95% was applied to analyze the data. Significance was set at *p* ≤ 0.001 (**) and *p* ≤ 0.05 (*). The difference between patients and controls was assessed with the Wilcoxon‐Mann–Whitney test.

## 3 Results

### 3.1 Histology and Histoenzymology

**FIGURE 1:**
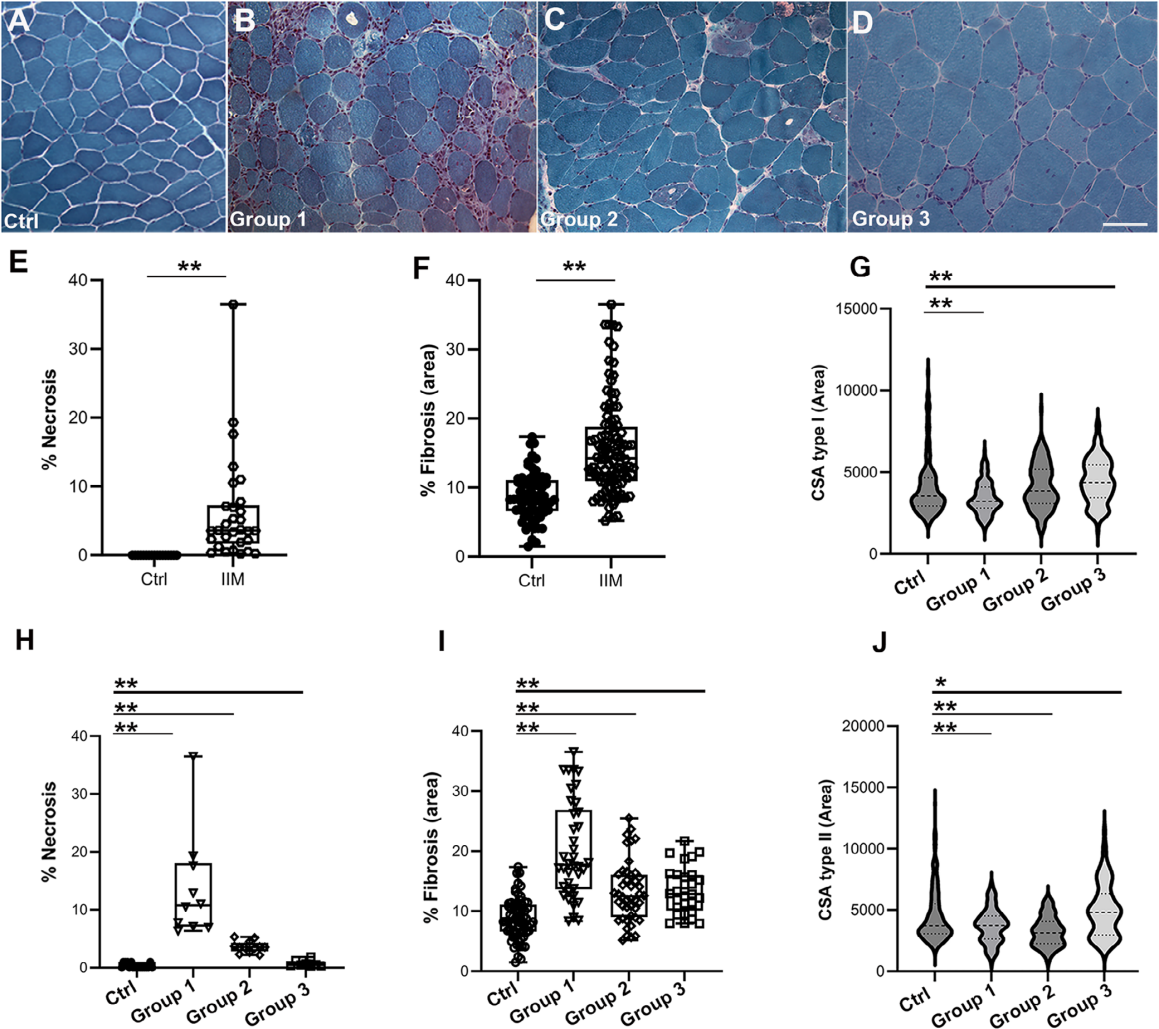
Representative MTG staining from control muscle (A) and from Group 1 (B), Group 2 (C) and Group 3 (D) patients. Scale bar 50 μm, magnification 20×. Box plots of necrotic fibres in controls and in all patients (E) and in the three groups separately (H). Box plots of the percentage of fibrosis in controls and all patients (F) and in the three groups separately (I). Violin plot of CSA for type I (G) and type II (J) fibres in controls and in the groups of patients. *p* ≤ 0.001 (**) and *p* ≤ 0.05 (*). Box plots showing the median, lower and upper quartiles (box), minimum and maximum values (whiskers) and the single points.

Myopathic changes as fibre size variability, centronucleated and splitting fibres were present in all patients with variable degrees compared to controls (Figure 1A–D, Table 2).

The percentage of necrotic fibres was significantly increased in all patients (Figure 1E). More in detail, the percentage was 13.60 ± 2.91 in Group 1 (*p* ≤ 0.0001), 3.62 ± 0.28 in Group 2 (*p* ≤ 0.0001) and 0.69 ± 0.2 in Group 3 (*p* ≤ 0.0001) (Figure 1H).

The statistical analysis based on comparison between the three pathological groups highlighted significant differences for all comparisons (*p* ≤ 0.0001). Endo‐ and perimysial fibrosis was significantly increased in all patients (15.67% ± 0.65%; *p* ≤ 0.0001) compared to controls (8.73% ± 0.41%) (Figure 1F). Specifically, the percentage of connective tissue deposition was 20.14% ± 1.31% in Group 1 (*p* ≤ 0.0001), 13.19% ± 0.76% in Group 2 (*p* ≤ 0.0001), 13.49% ± 0.74% in Group 3 (*p* ≤ 0.0001) and only 8.73% ± 0.41% in the control group (Figure 1I). Statistical analysis between the three pathological groups highlighted a significant difference between Group 1 and Group 2 (*p* ≤ 0.0001) and between Group 1 and Group 3 (*p* = 0.0003).

The percentage of centronucleated fibres was higher, but not significantly different from controls, in all three Groups. Statistical intra‐group comparison disclosed a significant difference only between Group 1 and Group 2 (*p* = 0.0341). The percentage of centronucleated fibres found in the muscles from control subjects (3.85 ± 0.67) still falls within the percentage considered normal (3%–5%). The percentage of splitting fibres was significantly increased in all patients compared to controls (0.144 ± 0.09), similar values being found in Group 1 (1.94 ± 0.33, *p* ≤ 0.0001) and Group 2 (1.85 ± 0.22, *p* ≤ 0.0001) and slightly less in Group 3 (0.67 ± 0.19, *p* = 0.0006). Significant differences between Group 1 and Group 3 (*p* = 0.0007) and between Group 2 and Group 3 (*p* ≤ 0.0001) emerged after statistical intra‐group comparison. Quantification of cross‐sectional area showed a significant reduction of type I fibre CSA in Group 1 (3427 μm^2^ ± 82 μm^2^, *p* = 0.0008), no significant changes for Group 2 (4121 μm^2^ ± 107 μm^2^, *p* = 0.0922) and a significant increase in Group 3 (4463 μm^2^ ± 126 μm^2^, *p* = 0.0002) compared to age‐matched controls (4096 μm^2^ ± 96 μm^2^) (Figure 1G). For type II fibre CSA, a significant reduction was detected in both Group 1 (3714 μm^2^ ± 112 μm^2^, *p* = 0.0094) and Group 2 (3242 μm^2^ ± 105 μm^2^, *p* ≤ 0.0001) compared to age‐matched controls (4496 μm^2^ ± 123 μm^2^), while a significant increase was detected in Group 3 (4984 μm^2^ ± 226 μm^2^, *p* = 0.0362) (Figure 1J). When statistically comparing the three pathological groups, significant differences were found for all analyses both for type I and type II fibres (see Table 2 for details). Lack or reduction of COX activity was detected in a variable number of fibres in 18 patients, results being similar in all three Groups, namely 6.35 ± 1.54 in Group 1, 6.42 ± 1.11 in Group 2 and 5.88 ± 1.50 in Group 3. Acid phosphatase activity was variably increased in 27 patients. More specifically, acid phosphatase activity was detected at the cytoplasmic level in 14 patients and at the interstitial level in the remaining cases. The percentage of acid phosphatase‐positive fibres was significantly higher in Group 1 (3.54 ± 0.619, *p* ≤ 0.0001) and in Group 2 (1.31 ± 0.391, *p* = 0.0055). After comparing the three pathological groups, statistically significant differences emerged between Group 1 and Group 2 (*p* = 0.0003) and between Group 1 and Group 3 (*p* ≤ 0.0001).

**TABLE 2:** Muscle biopsy profiles of patients.

Muscle biopsy	Group 1	Group 2	Group 3	Total	Ctrl	1 vs. 2	1 vs. 3	2 vs. 3
						*p*	*p*	*p*
% Necrosis	**13.60 ± 2.91** *p* ≤ 0.0001	**3.62 ± 0.28** *p* ≤ 0.0001	**0.69 ± 0.20** *p* ≤ 0.0001	**6.17 ± 1.37** *p* ≤ 0.0001	**0**	**≤ 0.0001**	**≤ 0.0001**	**≤ 0.0001**
% Area of fibrosis	**20.14 ± 1.31** *p* ≤ 0.0001	**13.19 ± 0.77** *p* ≤ 0.0001	**13.49 ± 0.74** *p* ≤ 0.0001	**15.67 ± 0.65** *p* ≤ 0.0001	8.73 ± 0.41	**≤ 0.0001**	**0.0003**	0.6276
Calibre variability								
Marked	4 (40)	4 (33.3)	3 (42.8)	11 (36.6)	0			
Moderate	5 (50)	5 (41.6)	1 (14.2)	11 (36.6)	0			
Mild	1 (1)	2 (16.6)	3 (42.8)	6 (20)	4 (22%)			
% Centronuclear fibres	5.29 ± 0.78 *p* = 0.1256	3.97 ± 0.87 *p* = 0.780	5.07 ± 1.1 *p* = 0.567	4.71 ± 0.53 *p* = 0.481	3.85 ± 0.67	**0.0341**	0.5279	**0.339**
% Splitting fibres	**1.94 ± 0.33** *p* ≤ 0.0001	**1.85 ± 0.22** *p* ≤ 0.0001	**0.67 ± 0.19** *p* = 0.0006	**1.55 ± 0.1** *p* ≤ 0.0001	0.144 ± 0.09	0.7171	**0.0007**	**≤ 0.0001**
CSA type I (area μm^2^)	**3427 ± 82** *p* = 0.0008	4124 ± 107 *p* = 0.0922	**4463 ± 126** *p* = 0.0002	3988 ± 64 *p* = 0.385	4096 ± 96	**≤ 0.0001**	**≤ 0.0001**	**0.0416**
CSA type II (area μm^2^)	**3714 ± 112** *p* = 0.0094	**3242 ± 105** *p* ≤ 0.0001	**4984 ± 226** *p* = 0.0362	**3858 ± 87** *p* = 0.0007	4496 ± 123	**0.0029**	**≤ 0.0001**	**≤ 0.0001**
COX deficit fibres (partial and total)	**6.35 ± 1.54** (*N* = 5) *p* ≤ 0.0001	**6.42 ± 1.11** (*N* = 9) *p* ≤ 0.0001	**5.88 ± 1.50** (*N* = 4) *p* ≤ 0.0001	**6.28 ± 0.78** (*N* = 18) *p* ≤ 0.0001	0	0.8307	0.8408	0.6821
PA positivity (fibres)	**3.54 ± 0.619** (*N* = 8) *p* ≤ 0.0001	**1.31 ± 0.391** (*N* = 5) *p* = 0.0055	0.14 ± 0.097 (*N* = 2) *p* = 0.2243	**1.71 ± 0.291** (*N* = 14) *p* = 0.0004	0	**0.0003**	**≤ 0.0001**	0.0398

*Note:* Data are presented as mean ± standard error, (% frequency). Significant *p*‐values (*p* ≤ 0.001 or *p* ≤ 0.05) are highlighted in bold.

Abbreviations: AP, acidic phosphatase; COX, cytochrome c oxidase; CSA, cross‐sectional area.

We classified fibre size variability as marked, moderate or mild; the results are reported in Table 2 along with muscle biopsy profiles of patients.

### 3.2 Analysis of Inflammatory Milieu

**FIGURE 2:**
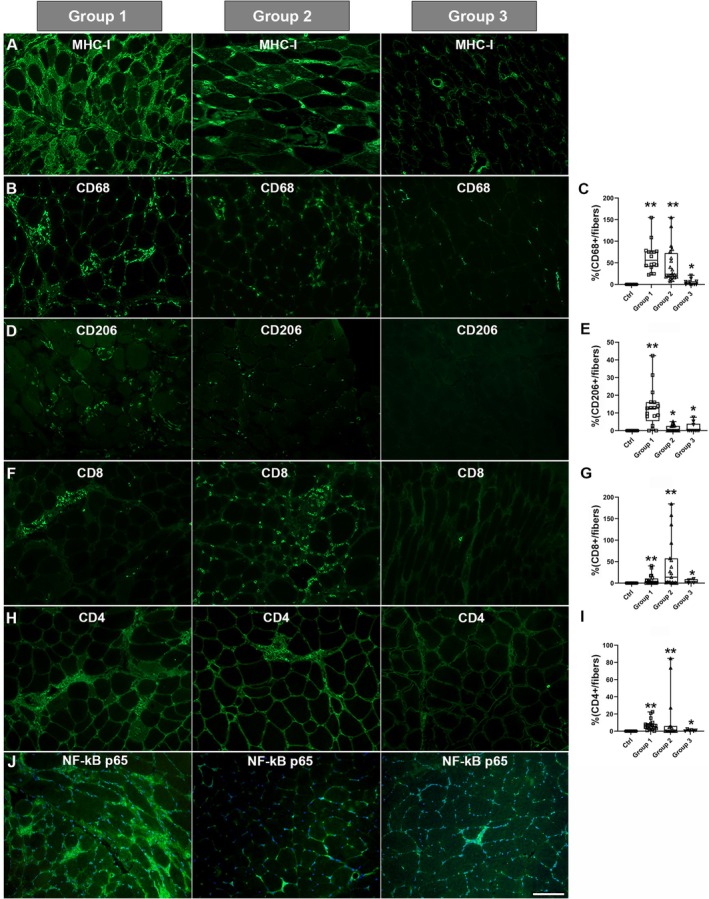
Representative immunostaining for MHC‐I (A), CD68 (B), CD206 (D), CD8 (F), CD4 (H) and NF‐kB p65 (J) for the three groups of patients. Scale bar 50 μm, magnification 20×. (C–I) Histogram of CD counts in the three groups of patients and controls. *p* ≤ 0.001 (**) and *p* ≤ 0.05 (*). Box plots showing the median, lower and upper quartiles (box), minimum and maximum values (whiskers) and the single points.

MHC‐I signal positivity was detected at the sarcolemmal and/or intracytoplasmic level in a high number of fibres in all tested patients, the signal intensity progressively decreasing from Group 1 to Group 3 patients (Figure 2A). CD68 and CD206 positivity displayed a similar trend in the three groups; indeed, greater positivity was seen in Group 1 patients, followed by those in Groups 2 and 3 (Figure 2B,C). CD68 positivity quantification was 62.6% ± 8.58%, 45.57% ± 9.42% and 5.19% ± 2.06% in Groups 1, 2 and 3 respectively (Figure 2F). CD206 positivity was significantly increased in Group 1 subjects (12.8% ± 2.68%, *p* ≤ 0.0001), lower values being found in Group 2 (1.30% ± 0.42%, *p* = 0.009) and Group 3 (1.63% ± 0.88%, *p* = 0.036) subjects (Figure 2C). Fluorescence staining for CD8 and CD4 showed a more intense signal in patients from Group 2 followed by those from Group 1 and, finally, by those from Group 3 in which only a few spots of positivity were observed (Figure 2D,E). In detail, CD8 positivity quantification reached 41.82% ± 13.5% in Group 2, 7.37% ± 2.64% in Group 1 and 4.45% ± 2.37% in Group 3 (Figure 2H), whereas CD4 positivity quantification showed 13.3% ± 7.15% in Group 2, 7.02% ± 1.38% in Group 1 and only 0.958% ± 0.39% in Group 3 (Figure 2I).

Statistical comparison of the three pathological groups highlighted significant differences between the three groups for CD68, between Group 1 vs. Group 2 (*p* ≤ 0.0001) and Group 1 vs. Group 3 (*p* = 0.0004) for CD206. Significant data were detected for Group 1 vs. Group 2 (*p* = 0.0342) for CD8, and for Group 1 vs. both Group 2 (*p* = 0.0411) and vs. Group 3 (*p* = 0.0015) for CD4.

Immunofluorescence analysis of the inflammatory marker NF‐kB p65 revealed positive staining in blood vessels, inflammatory cells, and muscle fibres, with varying intensity among patients. Our results suggest that p50/p65 activation is a general feature of IIM, regardless of patient subtype. However, we observed a more pronounced intensity and broader distribution of the signal in patients from Group 1, where positivity was particularly associated with mononuclear infiltrating cells, vessel walls and the cytoplasm of atrophic fibres.

Data about inflammatory milieu composition is summarised in Table S1.

**FIGURE 3:**
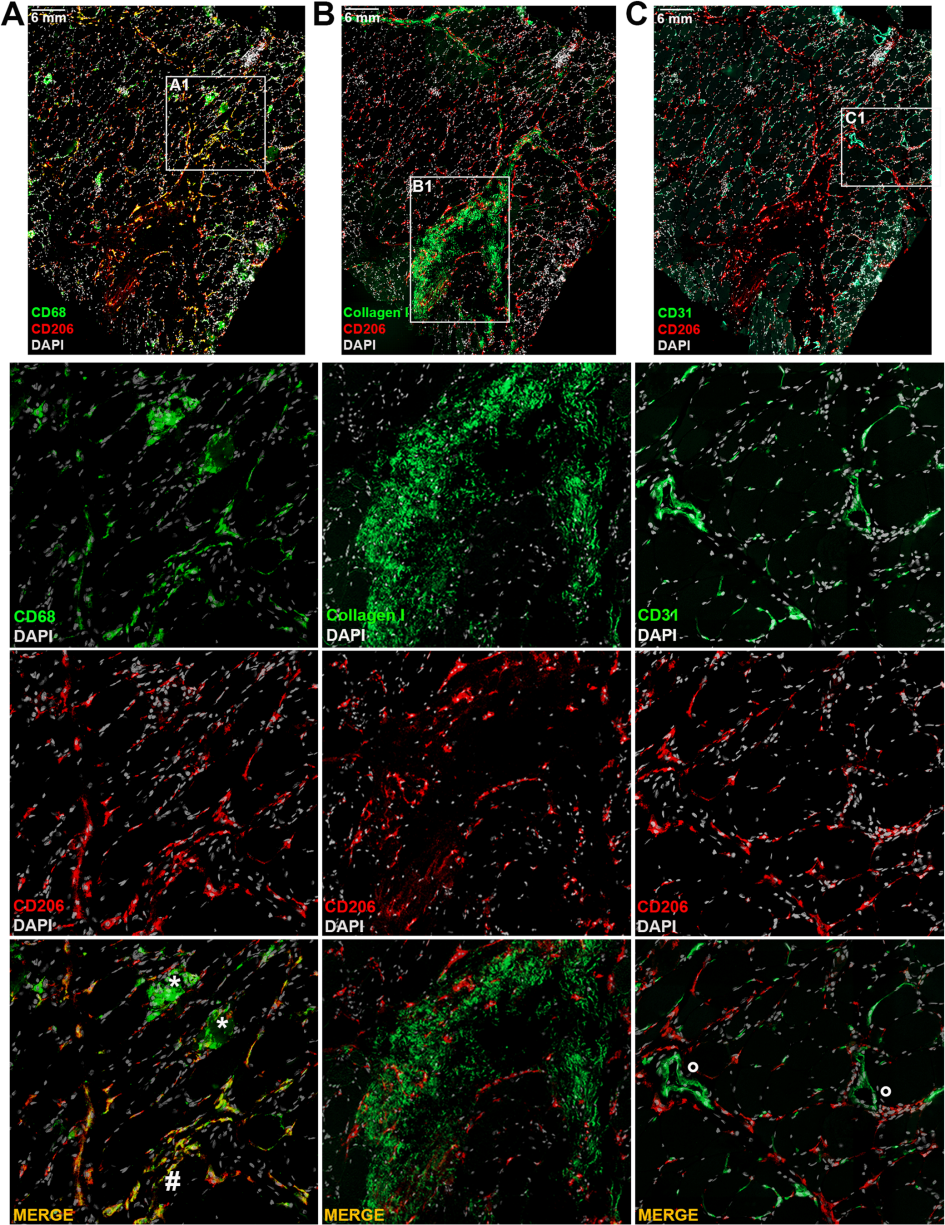
Representative immunostaining of muscle tissue from a Group 1 patient with MACSima platform. Scale bar 6 mm. Whole muscle sections were stained with CD206‐CD68‐DAPI (A), CD206‐collagen type I‐DAPI (B), and CD206‐CD31‐DAPI (C). Panels A1, B1 and C1 provide detailed magnified views of the muscle sections, displaying single‐channel images as described below. Graphic symbols indicate necrotic fibres (*), areas of colocalization (#), and vascular structures (°).

To better characterise CD206 positivity an experiment of spatial proteomics was performed on a selected patient from Group 1 in which the presence of CD206 positive cells was more evident. The cryostat muscle section was simultaneously incubated with CD206, CD68 (inflammatory), CD31 (vessels) and collagen type I (fibrosis) on the MACSima platform. The fluorescence signal for CD206 and CD68 revealed the presence of two distinct populations distributed throughout the sample. A marked positivity for CD68 was observed within necrotic fibres, where the signal for CD206 was absent. In contrast, a significant overlap of the two signals was evident in interfibrillar regions. The collagen I stain, whose increased expression is a hallmark of fibrotic processes, revealed the presence of a substantial number of CD206‐positive cells within the extracellular matrix. Considering the pro‐fibrotic characteristics of CD206‐positive cells, it can be hypothesised that the presence of CD206‐positive cells in regions of heightened collagen I deposition may actively contribute to the pathological accumulation of type I collagen. This hypothesis aligns with the known roles of CD206‐positive cells in driving fibrosis through the extracellular matrix. The co‐staining of CD206 and CD31, an established endothelial cell marker, revealed a significant presence of CD206‐positive cells near blood vessels, as observed in the detailed merged image. This spatial association suggests that CD206‐positive cells may actively contribute to angiogenic processes, particularly given their capacity to produce and release pro‐angiogenic factors such as VEGF. These findings further underscore the multifaceted role of CD206‐positive cells in modulating both fibrotic and vascular remodelling pathways (Figure 3).

### 3.3 Evaluation of Capillary Network Showed Altered Markers in Patients of Group 1

**FIGURE 4:**
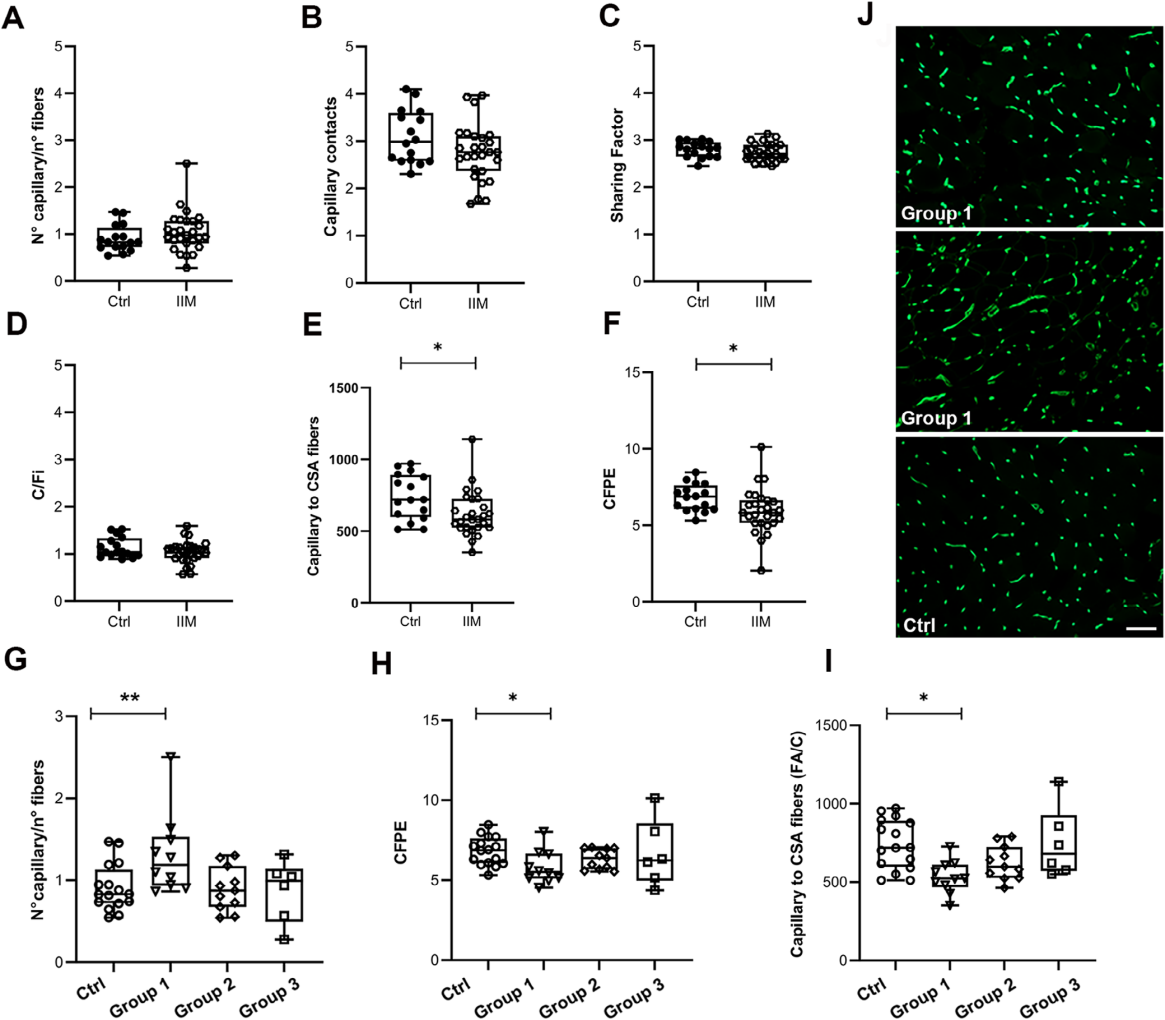
(A–F) Box plots for capillary network parameters in all patients and controls. (G) Box plots of *n*° of capillary/*n*° of fibres show a significant increase in Group 1. (H): Box plots of capillary‐to‐fibre perimeter exchange index (CFPE) show a significant reduction in Group 1. (I) Box plots of capillary to CSA (FA/C) show a significant reduction in Group 1. *p* ≤ 0.001 (**) and *p* ≤ 0.05 (*). (J) Representative image of Ulex staining in two patients from Group 1 and one control. Scale bar 50 μm. Box plots show the median, lower and upper quartiles (box), minimum and maximum values (whiskers) and the single points.

The evaluation of capillary network comparing all patients and controls showed no significant differences for almost all evaluated parameters (Figure 4A–D). Significant differences were detected for the ratio of capillaries to fibre CSA (Figure 4E), and for capillary‐to‐fibre perimeter exchange index (CFPE) (Figure 4F), both significantly reduced in patients compared to controls.

When patients were analysed in clusters, we detected a significant increase in the number of capillaries per fibre in Group 1 subjects (1.31 ± 0.15, *p* = 0.0087) compared to controls (0.91 ± 0.07) (Figure 4G), a significant reduction of CFPE in patients from Group 1 (5.81 ± 0.33, *p* = 0.0103) compared to controls (6.82 ± 0.22) (Figure 4H) and another significant reduction in capillaries to fibre CSA in patients from Group 1 (535.35 ± 33.31, *p* = 0.0028), compared to controls (740.13 ± 29.67) (Figure 4I). Intra‐group statistical analysis revealed a significant difference between Group 1 and Group 2 for the capillaries per fibre (*p* = 0.0197) and between Group 1 and Group 3 for capillary to fibre CSA (*p* = 0.0225). Data regarding the vascular network are summarised in Table S2.

### 3.4 Immunofluorescence of EndoMT Markers Showed Increased Mesenchymal Markers in Patients of Group 1

**FIGURE 5:**
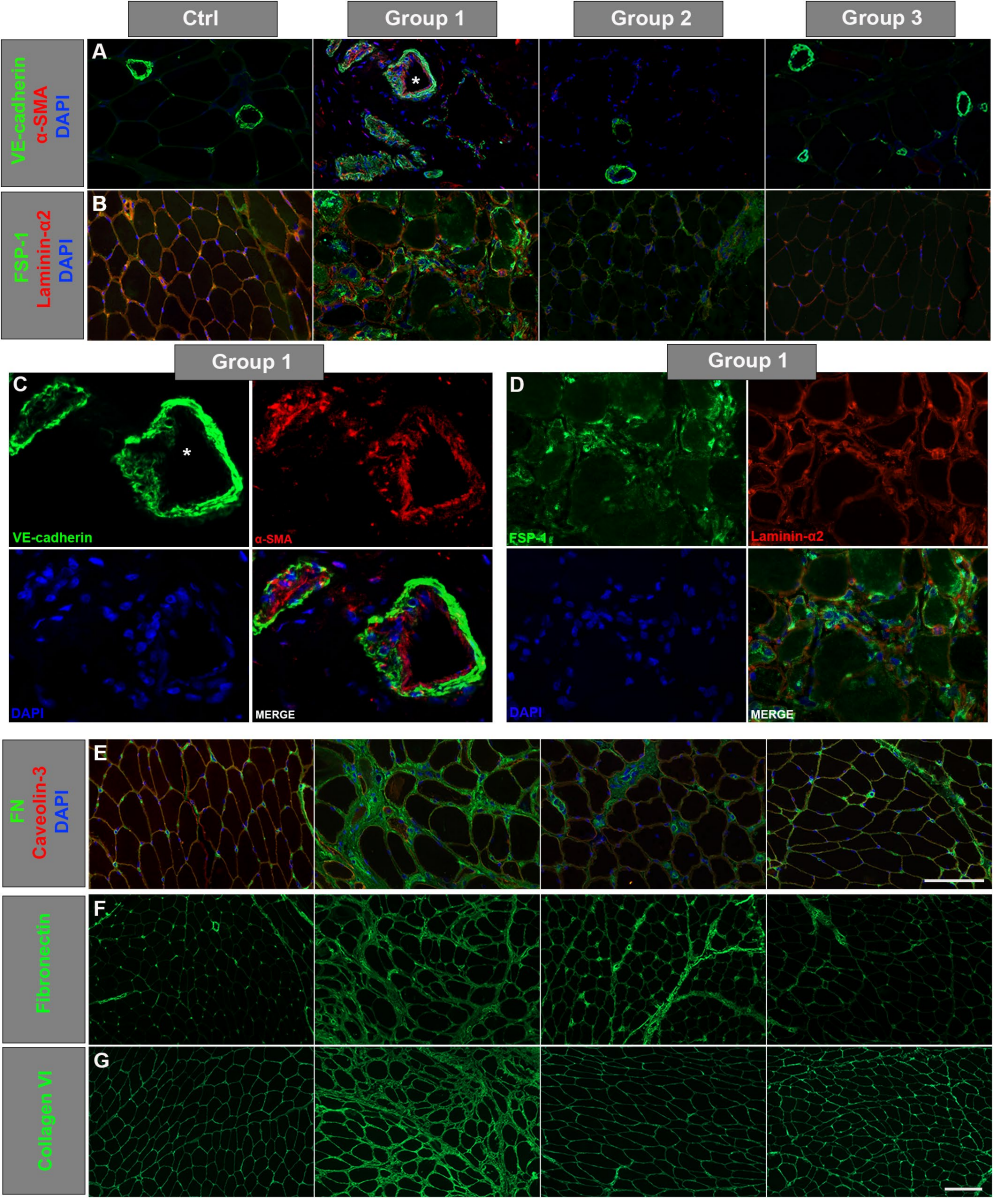
Representative immunofluorescence staining for EndoMT markers: VE‐cadherin (A), FSP‐1 (B). Panel (C) illustrates a single‐channel representation of a magnified vessel (*) stained with VE‐cadherin and α‐SMA, to emphasise the distribution of these markers. Panel (D) depicts a single‐channel staining, specifically identifying FSP‐1‐positive cells within the sample. (E) Representative immunofluorescence staining of FN in controls and in patients from the three different groups. Caveolin‐3 and laminin‐α2 for membrane staining, DAPI for counterstaining of nuclei. Scale bar: 50 μm, magnification 40×. (F, G) Single staining for fibronectin (F) and collagen VI (G) in patients and controls. FN, fibronectin; FSP‐1, fibroblast protein‐1. Scale bar: 50 μm, magnification 20×.

The staining patterns for EndoMT markers revealed distinct differences across the three Groups. We performed this evaluation only on a selected number of biopsies (*N* = 3 patients from each Group). Specifically, VE‐cadherin staining, a marker of endothelial cells and junctions, evidenced increased thickness and tortuosity of vessel walls in patients from Group 1 in which vessels often appeared disorganised, discontinuous, and less structurally cohesive. Also, we detected a fragmentation in VE‐cadherin signal suggesting a potential disruption of endothelial junctions (Figure 5A, and panel C). In contrast, patients from Groups 2 and 3 exhibited VE‐cadherin staining patterns indicative of more regular and organised vessels, closely resembling those observed in controls (Figure 5A). The α‐SMA staining pattern associated with the vessel walls demonstrated robust positivity exclusively in patients from Group 1, with no detectable α‐SMA positivity in patients from Groups 2 and 3 or in controls (Figure 5A).

FSP‐1 and FN, two markers of mesenchymal differentiation in EndoMT, tested in double staining with sarcolemmal markers, were more markedly increased in patients from Group 1 (Figure 5B,E). A moderate increase was observed in Group 2 patients, whereas Group 3 showed no significant alterations, mirroring control samples. These findings emphasise a gradient of EndoMT marker expression across the groups, Group 1 displaying the most pronounced abnormalities. In a patient from Group 1, the staining for FSP‐1‐positive cells was localised both in the interstitial space, in association with mononuclear cells, and at the sarcolemmal level; also, a diffuse signal was detected in the cytoplasm of some fibres, mainly those in degeneration (Figure 5B).

Marked deposition of FN was seen in the extracellular matrix (endo‐ and perimysium) in patients from Group 1 and, to a lesser extent, from Group 2 (Figure 5E). A slight increase in FN deposition was observed in Group 3 (Figure 5E). Single staining for fibronectin (Figure 5F) and collagen VI showed a marked deposition of both ECM proteins in patients from Group 1 (Figure 5G).

### 3.5 Western Blot Analysis

**FIGURE 6:**
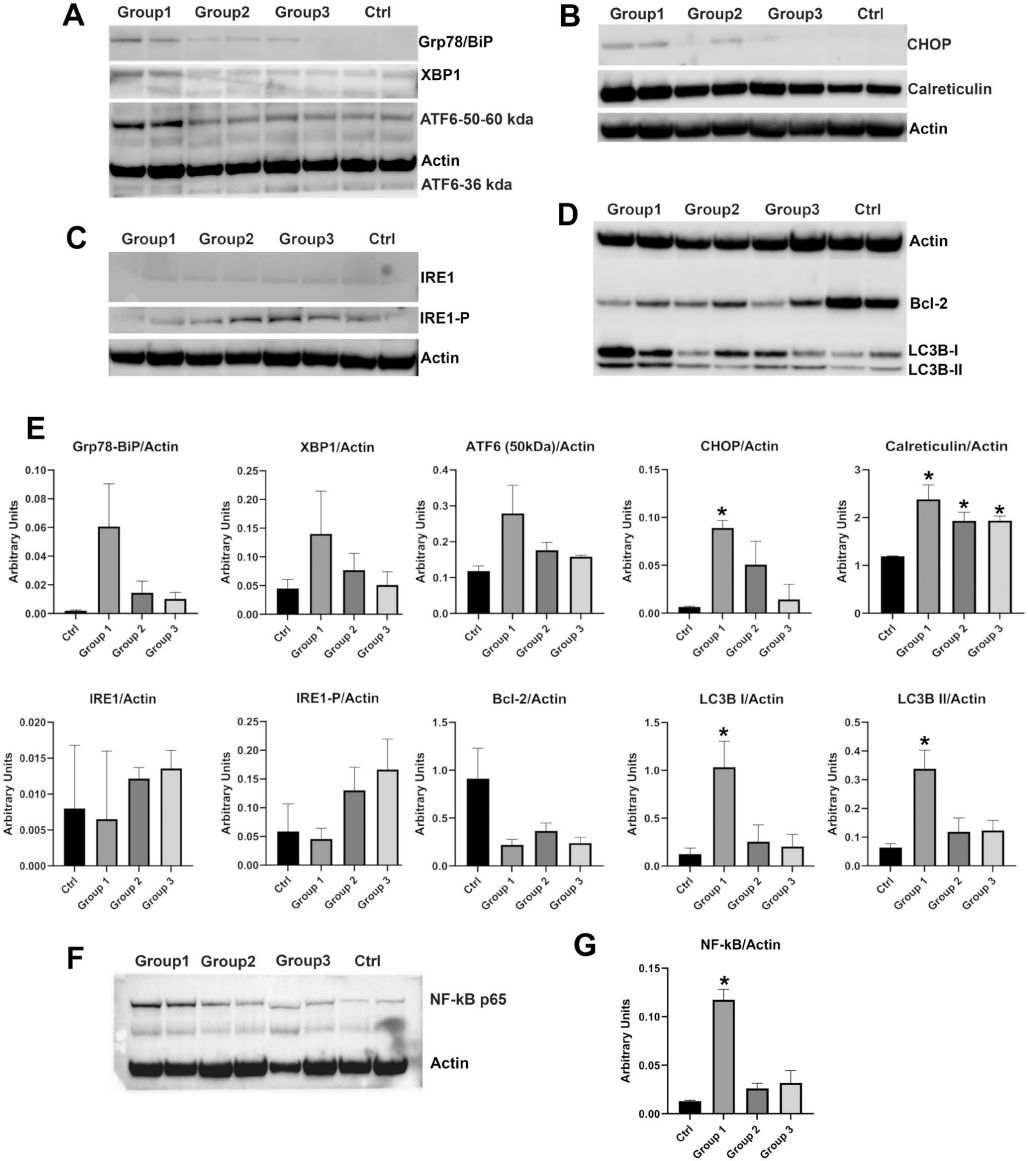
(A–D) Representative Western blot of ER stress, apoptotic and autophagic markers in patients and controls. (E) Western blot densitometry. The bar graph of protein densitometry shows a significant increase in calreticulin expression in all three groups compared to controls and significantly increased CHOP, LC3B‐I and LC3B‐II in patients from Group 1. *p* ≤ 0.05 (*). (F) Representative Western blot of NF‐kB p65 in patients and controls. (G) Western blot densitometry of NF‐kB p65 shows a significant increase in patients from Group 1.

Western blot analysis of ER stress markers, performed on a selected number of patients (*N* = 2) from each group, showed a different behaviour among the three groups of patients, more markedly so in those belonging to Group 1. Indeed, more evident bands for Grp78/BiP, XBP1, ATF6 and CHOP were detected in Group 1 subjects; also, only in Group 1 did the activation of the ATF6 branch show increased expression of a 50 kDa band and the presence of a faint band at 36 kDa, corresponding to the nuclear activated form of ATF6 (Figure 6A–C). Densitometric analysis of immunobands in Group 1 showed an increased expression of Grp78/BiP with concomitant increased signal for XBP1 and the activation of the ATF6 branch characterised by increased expression of 50 kDa and by the presence of a faint band at 36 kDa, corresponding to the nuclear activated form of ATF6.

CHOP, the final downstream protein in the ER stress signalling pathway, was significantly increased only in patients from Group 1 (0.089 ± 0.005, *p* = 0.0045) compared to controls (0.0065 ± 0.0004).

Western blot for IRE‐1 showed very faint bands in both patients and controls. The phosphorylated IRE‐1 bands showed a tendency to increase in Group 2 and Group 3 patients compared to those from Group 1. Western blot for calreticulin, a lectin‐associated chaperone, showed a general increase in protein expression in all patients compared to controls, with a more marked increase in Group 1 (Figure 6B). Densitometric analysis recorded the highest increase in Group 1 (2.38 ± 0.210, *p* = 0.037), similar expression in Groups 2 (1.93 ± 0.126, *p* = 0.0274) and 3 (1.94 ± 0.064, *p* = 0.0074), and lower values in controls (1.19 ± 0.0073).

Bcl‐2, a mitochondrial membrane protein that blocks apoptosis, was decreased in all patients compared to controls, even with great intra‐group variability, whereas the lipidated form of LC3B, LC3B‐II, which is generated during autophagosome formation, was highly increased in patients from Group 1 (1.033 ± 0.19 for LC3B‐I and 0.338 ± 0.045 for LC3B‐II) compared to controls (0.126 ± 0.044; *p* = 0.0438 for LC3B‐I and 0.0638 ± 0.0096; *p* = 0.028 for LC3B‐II) (Figure 6D).

Finally, the evaluation of NF‐kB showed increased expression in patients from all three groups, more marked and significantly in Group 1 (0.117 ± 0.011, *p* = 0.0108), and similar for Group 2 (0.026 ± 0.005, *p* = 0.1352) and Group 3 (0.032 ± 0.0126, *p* = 0.2720) compared to controls (0.013 ± 0.0008).

### 3.6 Immunofluorescences of ER Stress and Autophagy Markers

**FIGURE 7:**
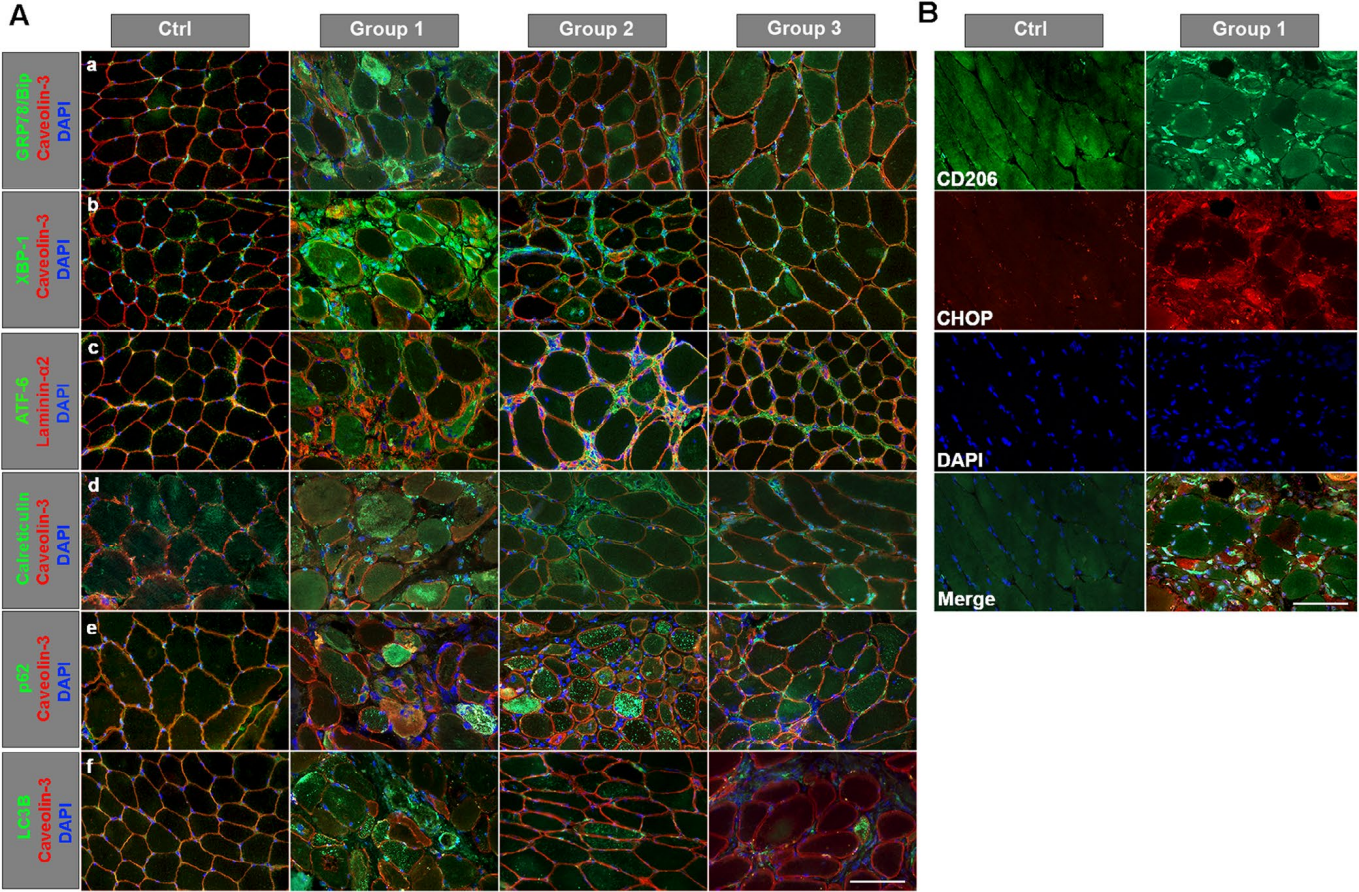
Representative immunofluorescence staining of ER and autophagy markers. Panel (A): GRP78/BiP (a), XBP1 (b), ATF6 (c), calreticulin (d), p62 (e) and LC3B (f) in controls and in patients from the three groups. Caveolin‐3 and laminin‐α2 for membrane staining, DAPI for counterstaining of nuclei. Panel (B): Co‐staining of CD206 and CHOP in one control and one Group 1 patient. Scale bar: 50 μm, magnification 40×.

GRP78/BiP staining showed a marked activation in patients from Group 1 with strong positivity in the cytoplasm of some fibres, more evident in necrotic fibres. Also, in patients from Group 1, a strong GRP78/BiP signal was associated with inflammatory cells. In patients from Group 2, GRP78/BiP positivity was observed in mononuclear cells and, at a lower intensity, in the cytoplasm of some fibres (Figure 7A). Similar results were observed for XBP1 with high activation in patients from Group 1—strong signal on immune cells and in the areas of cell infiltration, but also at the cytoplasm of some fibres—and progressively less intense staining in patients from the other two Groups (Figure 7B). P‐PERK signalling showed increased positivity in patients from Group 1 in the cytoplasm of suffering fibres and in the endomysial connective tissue next to cellularity (Figure 7C). In patients from Group 1, ATF6 positivity was mainly located at the perimysium, in immune cells and in some fibres probably undergoing myophagocytosis as previously reported [23]. In patients from Group 2, positivity was observed in perimysium and in few fibres. In patients from Group 3 signalling was mainly localised at perimysium even if a punctate cytoplasmic positivity was observed in few fibres (Figure 7D).

Calreticulin staining showed a diffusely increased cytoplasmic signal in all patients, particularly marked in patients from Group 1 in which a punctiform positivity in the cytoplasm of both normal and affected fibres was observed (Figure 7E). In Group 1, p62 positivity was observed in the cytoplasm of scattered necrotic fibres. P62 staining was increased in patients from Group 2, with a prevalence of punctate staining, sometimes with the tendency to form cytoplasmic aggregates with variable intensity including fibres with a small punctata staining, fibres with increased density of the puncta and fibres with a strong staining in the entire cytoplasm. In patients from Group 3, p62 staining appeared as small puncta in some fibres. These data suggested that the p62‐mediated autophagy pathway is activated differently in the three different patients' groups (Figure 7F).

LC3B staining showed a marked increase of punctate signals in the cytoplasm of several fibres from Group 1 patients. The punctate signal appeared as smaller dots scattered in the cytoplasm of a smaller number of fibres in both Group 2 and Group 3 patients (Figure 7G).

The immunofluorescence co‐staining of CHOP and CD206 showed increased expression of CHOP that partially colocalized with CD206‐positive cells in patients from Group 1 (Figure 7 Panel B). In patients from Groups 2 and 3 no CHOP signal was detected similarly to what was observed in controls (data not shown). We performed this evaluation only on a selected number of biopsies (*N* = 3 patients from each Group).

## 4 Discussion

In our study, we investigated the intricate relationship between inflammation, vascular network dynamics, and endoplasmic reticulum (ER) stress in a cohort of patients diagnosed with idiopathic inflammatory myopathies (IIMs). All examined patients had markedly elevated serum creatine kinase levels and presented classical clinical symptoms such as muscle weakness, myalgias, and fatigue. To date, the immunopathological mechanisms underlying this group of conditions are still not fully understood.

In cases of IIM, the degree of muscle inflammation does not correlate with the severity of the disease or with the morphological changes observed in muscle biopsies. This indicates that non‐immunological factors, i.e., ER stress and disruptions in protein responses like the unfolded protein response (UPR) and autophagy, may play a significant role.

Some of these aspects have been investigated in our patients. Their muscle biopsies exhibited the typical morphological changes of IIMs, including increased variability in fibre size, fibre splitting, and centronucleated fibres [24, 25]. Histological evaluation of muscle biopsies highlighted significant differences in the presence of necrotic fibres and in fat and connective tissue deposition.

The clustering of our patients in three groups based on the percentage of necrotic fibres in their muscle biopsy allowed us to identify interesting differences, the most significant ones concerning the composition of the inflammatory milieu, the ER activation, the vascular network and fibrosis.

In muscle biopsies from Group 1 patients, those with the highest percentage of necrotic fibres, we observed the most severe dystrophic changes including a marked increase in fibrosis and a significant reduction in cross‐sectional area (CSA) of both type I and type II fibres. The same patients showed a more marked inflammatory milieu with evidence of CD68 macrophages and CD206 positive cells. CD206 positive cells, an indicator of alternatively activated M2 macrophages, are typically found in a necrotizing environment [26] and are pivotal in tissue repair and in remodelling processes playing a critical role in promoting angiogenesis [27, 28] and in resolving inflammation [29, 30].

Chronic muscle inflammation can impact the microvascular system, including capillaries as reported [30, 31]. The exact nature of these changes may vary based on the specific type of inflammatory myopathy and individual patient characteristics. In our study, patients from Group 1 showed a reduced capillary‐to‐fibre perimeter exchange index that may compromise oxygen and nutrient supply to muscle fibres, contributing to muscle atrophy, and a significantly increased capillary density. This change may be considered an attempted compensatory response to the enhancing request for oxygen and nutrient delivery to affected muscle tissue and/or the reaction to the presence of numerous CD206‐positive cells producing pro‐angiogenetic conditions via the secretion of VEGF, IL 10, and TGF‐β [32, 33].

The high amount of CD206 positive cells may contribute to the activation of the Endothelial‐to‐Mesenchymal Transition process (EndoMT) and to the vascular remodelling. EndoMT represents a multifaceted process characterised by loss of cell–cell junctions, acquisition of invasive and migratory properties, loss of endothelial markers and gain of mesenchymal markers. Under conditions of chronic inflammation, sustained activation of endothelial cells by inflammatory stimuli causes alterations in normal endothelial function. Inflammation has a key role in inducing pathological EndoMT in chronic pulmonary arterial hypertension [34], atherosclerosis [35], metabolic syndromes [36], cardiovascular diseases [37], cancer [38] and several fibrotic conditions [39, 40, 41].

The increased expression of the mesenchymal markers FSP‐1 and fibronectin, as well as the positivity for α‐SMA associated with vessels, observed in patients from Group 1, suggests an activation of the EndoMT process. In the same patients, the vessel walls appeared frequently discontinuous and less organised.

Chronic inflammation is closely linked to ER stress. It is known that ER stress and UPR activation are prominent in patients with IIMs [23], and that several factors may contribute to ER stress, including oxidative stress, calcium dysregulation, and inflammation itself, as well as the presence of CD206‐positive M2 macrophages. The presence of high levels of CD206‐positive cells in Group 1 prompted us to evaluate the ER stress in our patients. Three different branches, PERK, IRE1‐ and ATF6‐mediated, orchestrate the UPR activation.

Patients from Group 1 showed an increased expression of Grp78/BiP, XBP1 and ATF6, indicating a more marked ER stress. Of the three UPR branches, the mechanism of ATF6 activation remains the least well understood. After activation and translocation to nuclei, ATF6 regulates the expression of several target genes, including BiP and CCAAT/enhancer‐binding protein homologous protein (CHOP) [42]. In patients from Group 1 we detected a significant increase in CHOP protein expression compared to other patients and to controls. Conversely, the activation of the IRE1 arm appears more evident in the patients from the other two Groups, suggesting a different ER stress not involving the expression of CHOP.

CHOP, which is physiologically ubiquitously expressed at very low levels, serves as a multifunctional transcription factor that also contributes to cellular activities including apoptosis [43, 44], autophagy [45], and inflammation [46, 47].

The ER stress response, acting to reestablish protein homeostasis, may also be involved in a maladaptive response because the IRE1, ATF6, and PERK pathways are also connected to pro‐apoptotic pathways by downregulation of the expression of pro‐apoptotic genes, such as Bcl‐2. In all our patients we detected a generalised reduction of Bcl‐2 expression; differently, a clear activation of LC3 was observed only in the patients from Group 1. A higher expression of CHOP is associated with the esterification capacity of LC3B and can promote the transformation of LC3B‐I to LC3B‐II [48] and autophagy activation.

CHOP has been implicated in the pathogenesis of various fibrotic diseases, including liver, pulmonary and cardiac fibrosis. In these conditions, chronic ER stress and CHOP activation contribute to the sustained activation of fibrotic pathways. Interestingly, CHOP upregulation is related to tissue fibrosis and to M2 macrophages polarisation [49]. CHOP deficiency attenuates the induction of M2 macrophages, thereby repressing TGF‐β1 secretion involved in lung fibrosis [49]. Similarly to patients with IPF, we also observed an increased expression of CHOP in Group 1 patients in association with the infiltrated macrophages CD206‐positivity cells.

In pulmonary fibrosis, increased CHOP expression was reported and related to myofibroblast activation [50], as in renal tubulointerstitial fibrosis [51]. Using a *CHOP* knockout mice model, these authors demonstrated that *CHOP* deficiency ameliorates lipid peroxidation, tubular apoptosis, and inflammatory milieu in the unilateral ureteral obstruction (UUO) model of renal fibrosis [51]. In a previous study on hepatic fibrosis in cholestatic liver injury, Tamaki N [52] demonstrated that *CHOP* deletion alleviated hepatocyte death and reduced hepatic fibrosis, with an inhibitory effect on TGF‐β1 induction and hepatic stellate cell activation [52]. Furthermore, ablation of *CHOP* can attenuate cardiac hypertrophy, cardiac dysfunction, and fibrosis with less evidence of apoptotic cell death and an attenuation of myocardial inflammation in mice subjected to a transverse aortic constriction [53, 54].

## 5 Conclusions

This study contributed to a redefined stratification of patients with idiopathic inflammatory myopathies (IIMs). Our findings revealed distinct inflammatory profiles among our patients, particularly influenced by the presence of CD206‐positive cells. Infiltration of CD206‐positive cells and increased CHOP expression were identified as the most prominent features distinguishing Group 1 patients from those belonging to Groups 2 and 3.

Our observations suggest that targeting CD206‐positive cells and modulating CHOP expression could potentially enhance the inflammatory response and mitigate fibrosis progression in these patients. These insights highlight the influence of cellular infiltrates on varied responses, contributing to our understanding of the molecular mechanisms underlying IIM pathogenesis.

The complex interaction between inflammation, ER stress, vascular remodelling and fibrosis in severe muscle pathology within the IIMs was particularly evident in Group 1 patients whose evidence of necrosis is more massive. These disease processes contribute to muscle atrophy—above all via the activation of CHOP [55, 56]—impaired function, and potentially adverse clinical outcomes.

In preclinical studies, inhibition or knockout of CHOP has shown promising results in reducing fibrosis in animal models. This suggests that targeting CHOP pharmacologically could be a potential strategy for treating fibrotic diseases and to attenuate ER stress. While CHOP inhibition shows potential therapeutic benefits, there are challenges in translating these findings into clinical practice. Further research is needed to fully understand the role of CHOP in different fibrotic diseases, as well as to develop safe and effective CHOP‐targeting therapies. In conclusion, CHOP is indeed a pharmacological target of interest for treating fibrosis due to its role in promoting fibrotic processes under stress conditions. Further elucidation of inflammatory infiltrate composition through spatial proteomics and transcriptomics studies may contribute to identifying downstream effectors of ER stress as therapeutic targets for regulating cell survival and fibrotic remodelling.

The stratification of patients based on necrotic fibre percentage has revealed distinct molecular profiles which suggest that IIMs are not a homogeneous group of disorders, but rather a spectrum of diseases with distinct molecular signatures. This scenario discloses a potentially new approach for more personalised treatment strategies, and it could become a useful instrument to understand and predict response to therapeutic protocols.

### 5.1 Limitations

The relatively small number of patients (*n* = 30) and controls (*n* = 18) may limit the generalizability of our findings. Larger cohorts are needed to validate and strengthen the observed associations, especially in subgroup analyses. The study is cross‐sectional in nature, which limits the ability to establish causal relationships between ER stress, inflammatory cell infiltration, vascular remodelling, and fibrosis progression. Longitudinal studies would be required to further clarify the alterations observed. Another limitation of this study is the fact that, in addition to the histological and molecular study conducted, it was not possible to make correlations with functional tests for the assessments of muscle performance (e.g., strength measurements, functional scales) as they were not available for a consistent number of patients. This limits the ability to correlate morphological and molecular findings with clinical severity or prognosis.

## Author Contributions

**Monica Sciacco:** conceptualization (equal), writing – original draft (equal), writing – review and editing (equal). **Patrizia Ciscato:** methodology (equal), visualization (equal). **Letizia Bertolasi:** methodology (equal), visualization (equal). **Maria Guttuso:** methodology (equal), visualization (equal). **Stefania Corti:** writing – review and editing (equal). **Deborah Mattinzoli:** methodology (equal), visualization (equal). **Masami Ikeata:** methodology (equal), visualization (equal). **Giuseppe Castellano:** methodology (equal), visualization (equal). **Giacomo Pietro Comi:** writing – review and editing (equal). **Simona Zanotti:** conceptualization (equal), data curation (equal), formal analysis (equal), investigation (equal), methodology (equal), writing – original draft (equal).

## Ethics Statement

The study was conducted in accordance with the Declaration of Helsinki and approved by the Institutional Ethics Committee of the ‘IRCCS Ca' Granda Foundation Ospedale Maggiore Policlinico, Italy’.

## Consent

Written informed consent has been obtained from both patient and control to publish this paper.

## Conflicts of Interest

The authors declare no conflicts of interest.

## Supporting information


**Tables S1–S2:** jcmm70919‐sup‐0001‐TablesS1‐S2.docx.

## Data Availability

The data presented in this study are available on reasonable request from the corresponding author.
