# Supplementary material for: The Role of C/EBP‐Homologous Protein in Idiopathic Inflammatory Myopathies
Source: J Cell Mol Med. 2025 Oct 29;29(21):e70919. doi: 10.1111/jcmm.70919 (PMC12571188; doi:10.1111/jcmm.70919)
Supplement: Supplementary file 1 — Tables S1–S2: jcmm70919‐sup‐0001‐TablesS1‐S2.docx. [file JCMM-29-e70919-s001.docx]

**Supplementary Tables**

***Table S1****. Inflammatory profiles for the patients of the three Groups*

| **Inflammatory milieu** | **GROUP 1** | **GROUP 2** | **GROUP 3** | 1 vs 2  p value | 1 vs 3  p value | 2 vs 3  p value |
| --- | --- | --- | --- | --- | --- | --- |
| **MHC1 (HLA) positivity** |  |  |  |  |  |  |
| Membrane positivity | 8 (80%) | 9 (81.8%) | 8 (100%) |  |  |  |
| Cytoplasm positivity | 2 (20%) | 1 (9.09%) | 0 |  |  |  |
| **% CD68** | **62.6 ± 8.58** p≤0.0001 | **45.57 ± 9.42** p≤0.0001 | **5.19 ± 2.06** p=0.0020 | **0.0449** | **≤0.0001** | **≤0.0001** |
| **%CD206** | **12.8 ± 2.68** p≤0.0001 | **1.30 ± 0.42** p=0.009 | **1.63 ± 0.88** p=0.036 | **≤0.0001** | **0.0004** | 0.9873 |
| **% CD8** | **7.37 ± 2.64** p≤0.0001 | **41.82 ± 13.5** p≤0.0001 | **4.45 ± 2.37** p=0.0041 | **0.0342** | 0.9704 | 0.2226 |
| **% CD4** | **7.02 ± 1.38** p≤0.0001 | **13.3 ± 7.15** p=0.0169 | **0.958 ± 0.39** p=0.0025 | **0.0411** | **0.0015** | 0.9665 |

*Data are presented as mean ± standard error, (%frequency). Significant P-values (p≤0.001 or p≤0.05) are highlighted in bold.*

***Table S2****. Vascular network characteristics for the patients of the three Groups*

| **Vascular network** | **GROUP 1** | **GROUP 2** | **GROUP 3** | **CTRL** | 1 vs 2  p value | 1 vs 3  p value | 2 vs 3 p value |
| --- | --- | --- | --- | --- | --- | --- | --- |
| % (n° capillary/n° fibers) | **1.31 ± 0.15** p=0.0087 | 0.89 ± 0.08 p=0.903 | 0.87 ± 0.15 p=0.971 | 0.91 ± 0.07 | **0.0197** | 0.1471 | 0.8075 |
| CC: capillary content | 2.78 ± 0.15 p=0.246 | 2.71 ± 0.19 p= 0.266 | 2.76 ± 0.31 p= 0.440 | 3.09 ± 0.14 | 0.7620 | 0.8749 | 0.7325 |
| C/Fi: capillary-to-fiber ratio | 1.06 ± 0.05 p=0.310 | 1.02 ± 0.07 p=0.367 | 1.04 ± 0.15 p=0.693 | 1.14 ± 0.05 | 0.8094 | 0.9087 | 0.6605 |
| CFPE: capillary-to-fiber perimeter exchange index | **5.81 ± 0.33** p=0.0103 | 6.32 ± 0.18 p=0.1341 | 6.70 ± 0.85 p=0.693 | 6.82 ± 0.22 | 0.0845 | 0.4278 | 0.9612 |
| SF: sharing factor | 2.69 ± 0.05 p=0.066 | 2.74 ± 0.05 p=0.400 | 2.79 ± 0.10 p=0.857 | 2.80 ± 0.04 | 0.5663 | 0.6131 | 0.8067 |
| FA/C: capillary to CSA fiber | **533.35 ± 33.31** p=0.0028 | 623.18 ± 32.46 p=0.078 | 747.63 ± 91.31 p=0.914 | 740.13 ± 29.67 | 0.0845 | **0.0225** | 0.311 |

*Data are presented as mean ± standard error, (%frequency). Significant P-values (p≤0.001 or p≤0.05)*

*are highlighted in bold.*
